# Glycosylation of immunoglobulin G determines osteoclast differentiation and bone loss

**DOI:** 10.1038/ncomms7651

**Published:** 2015-03-31

**Authors:** Ulrike Harre, Stefanie C. Lang, René Pfeifle, Yoann Rombouts, Sabine Frühbeißer, Khaled Amara, Holger Bang, Anja Lux, Carolien A. Koeleman, Wolfgang Baum, Katharina Dietel, Franziska Gröhn, Vivianne Malmström, Lars Klareskog, Gerhard Krönke, Roland Kocijan, Falk Nimmerjahn, René E. M. Toes, Martin Herrmann, Hans Ulrich Scherer, Georg Schett

**Affiliations:** 1 Department of Internal Medicine 3, University of Erlangen-Nuremberg, Erlangen 91054, Germany; 2 Nikolaus Fiebiger Center of Molecular Medicine, University of Erlangen- Nuremberg, Erlangen 91054, Germany; 3 Department of Rheumatology, Leiden University Medical Center, Leiden 2300 RC, The Netherlands; 4 Center of Proteomics and Metabolomics, Leiden University Medical Center, Leiden 2300 RC, The Netherlands; 5 Department of Chemistry and Pharmacy, Interdisciplinary Center for Molecular Materials (ICMM), University of Erlangen-Nuremberg, Erlangen 91054, Germany; 6 Rheumatology Unit, Department of Medicine, Karolinska Institute and Karolinska University Hospital, Solna 17177, Sweden; 7 Orgentec Diagnostika, Mainz 55129, Germany; 8 Department of Genetics, University of Erlangen-Nuremberg, Erlangen 91054, Germany

## Abstract

Immunglobulin G (IgG) sialylation represents a key checkpoint that determines the engagement of pro- or anti-inflammatory Fcγ receptors (FcγR) and the direction of the immune response. Whether IgG sialylation influences osteoclast differentiation and subsequently bone architecture has not been determined yet, but may represent an important link between immune activation and bone loss. Here we demonstrate that desialylated, but not sialylated, immune complexes enhance osteoclastogenesis *in vitro* and *in vivo*. Furthermore, we find that the Fc sialylation state of random IgG and specific IgG autoantibodies determines bone architecture in patients with rheumatoid arthritis. In accordance with these findings, mice treated with the sialic acid precursor N-acetylmannosamine (ManNAc), which results in increased IgG sialylation, are less susceptible to inflammatory bone loss. Taken together, our findings provide a novel mechanism by which immune responses influence the human skeleton and an innovative treatment approach to inhibit immune-mediated bone loss.

## Abstract (web summary)

The IgG sugar moiety modulates the binding of immune complexes to their Fcγ receptors resulting in pro- or anti-inflammatory response. This study shows that IgG sialylation also affects osteoclastogenesis and bone mass in mice and humans, identifying a new link between bone and the immune system.

## Graphical abstract

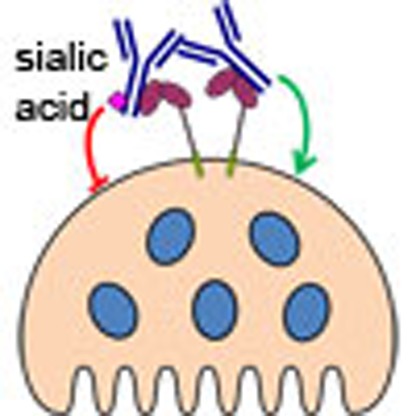

IgG is a potent effector protein of the humoral immune response. Besides its role in antigen binding, IgG has been well established to regulate the activity of immune cells by binding to activating or inhibitory FcγR, mediating key effector functions of IgG such as the clearance of pathogens or antibody-dependent cellular cytotoxicity. The binding of IgG to FcγR is influenced by the glycan attached to asparagine-297 in the Fc part of IgG^1^. This glycan is composed of a conserved heptamer backbone that consists of *N*-acetylglucosamine and mannose residues and is modified by the addition of several sugar moieties (Supplementary Fig. 1). Changes in IgG glycosylation are considered to result in an altered Fc conformation and affect the binding affinity of IgG to FcγR^2^,^3^. Hence, attachment of terminal sialic acid residues on IgG appears to mediate the anti-inflammatory effects of intravenous IgG^4^,^5^. Conversely, the absence of terminal sialic acid residues increases the affinity to activating FcγR^6^,^7^. The exact mechanism, by which these sugar moieties affect biological functions, is, however, not fully clarified^8^. Some^9^,^10^,^11^, but not all^12^, studies have suggested that anti-inflammatory effects of sialylated IgG are attributed to an enhanced binding of IgG to anti-inflammatory lectin receptors, such as the dendritic cell-specific intercellular adhesion molecule-3-grabbing non-integrin^9^ or the dendritic cell immunoreceptor^10^ according to changes in the Fc conformation.

Bone homeostasis is assured by a concerted action of bone forming osteoblasts and bone resorbing osteoclasts. This balance can be impaired by the immune system as it is observed in bone destructive autoimmune diseases such as rheumatoid arthritis. However, while cytokine-mediated effects have been extensively studied in the past^13^,^14^, there has been limited research regarding the impact of IgG or immune complexes on bone. Osteoclasts originate from the monocyte/macrophage lineage. Apart from specific growth factors and cytokines, such as the macrophage colony stimulating factor (M-CSF) and the receptor activator of NF-κB ligand (RANKL), osteoclastogenesis requires costimulatory signals from the immunoreceptor tyrosine-based activation motif-harbouring proteins, FcR common γ chain (FcRγ) and DNAX-activating protein (DAP)12 (refs 15, 16). In addition, osteoclasts express FcγR in comparable amounts to macrophages and dendritic cells^17^,^18^, suggesting that they may be affected by immune complexes. This notion is supported by a study showing that crosslinking of FcγR on murine preosteoclasts leads to higher osteoclast numbers^19^ and our previous finding that antibodies against citrullinated proteins (ACPA) directly promote osteoclastogenesis^20^. As pathogenic antibodies such as ACPA have been reported to contain less sialic acid in their Fc glycan than whole-blood IgG^21^, we further hypothesized that IgG sialylation may have an impact on immune complex–osteoclast interactions.

In this study, we used heat-aggregated IgG as an immune complex mimetic without antigen restrictions^22^ to investigate the effects of IgG sialylation on immune complex–osteoclast interactions. We found IgG sialylation to be a main regulator for the pro-osteoclastogenic potential of immune complexes as only non-sialylated immune complexes stimulated osteoclastogenesis *in vitro* as well as *in vivo*. In addition, we found higher Fc sialylation levels of both random IgG as well as specific autoantibodies to be correlated with higher bone volume in patients with RA. Administration of the sialic acid precursor ManNAc resulted not only in elevated sialylation levels of IgG, but also in a decreased susceptibility to collagen-induced arthritis (CIA)-mediated bone erosion in DBA/1J mice, suggesting a protective role of sialylated IgG against autoimmune-mediated bone loss.

## Results

### Desialylated immune complexes stimulate osteoclastogenesis

To investigate the effects of immune complexes on human osteoclast formation, we differentiated human monocytes in the presence of M-CSF, RANKL and TGF-β to the stage of mono- and bi-nucleated preosteoclasts and challenged them with 100 μg ml^−1^ of immune complex mimetics derived by heat aggregation of pooled human IgG. One fraction of the IgG had been enzymatically desialylated before aggregation (Supplementary Fig. 2) to determine the importance of IgG sialylation for immune complex-mediated effects on preosteoclasts. In addition, we used immune complexes that had been deglycosylated, and therefore lost their FcγR binding capacity, as a control for unspecific effects by the amount of protein added to the osteoclast culture. After 24 h of stimulation, staining for tartrate-resistant acidic phosphatase (TRAP) was performed and osteoclasts were counted.

**Figure 1:**
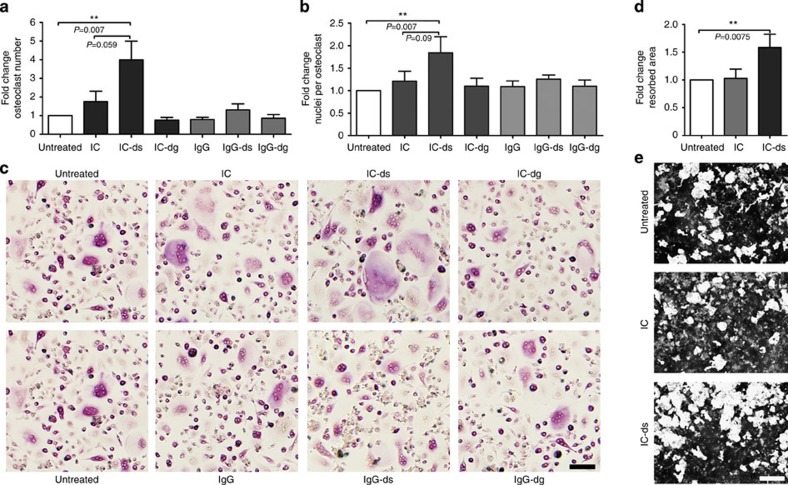
Fc-glycosylation determines the osteoclastogenic effects of human immune complexes. (**a**) Fold change of osteoclast number and (**b**) nuclei per osteoclast after 24 h treatment of human preosteoclasts with 100 μg ml^−1^ of native, desialylated (ds) or deglycosylated (dg) monomeric (IgG) or complexed (IC) pooled human IgG. TRAP-positive cells with ≥3 nuclei were considered as osteoclasts. Bars show mean±s.e.m. of five independent experiments. (**c**) Representative micro images. Scale bar, 100 μm. (**d**) Fold change of the resorbed area after 24 h treatment of human preosteoclasts with 100 μg ml^−1^ of native or desialylated (ds) IgG complexes (IC) in calcium phosphate-coated wells. Bars show mean±s.e.m. of five independent experiments (**e**) Representative micro images. Scale bar, 500 μm. Statistical analysis was performed with Mann–Whitney *U*-test. **P<0.01.

The stimulation with desialylated immune complexes (IC-ds) resulted in an accelerated osteoclastogenesis with an increase in osteoclast number, nuclei per osteoclast and bone resorption (Fig. 1). In contrast, after stimulation with untreated (sialylated) immune complexes (IC), we observed only a very weak tendency to higher osteoclast numbers and no difference in bone resorption. Neither the deglycosylated immune complexes (IC-dg) nor monomeric IgGs showed an effect, indicating a specific interaction between non-sialylated immune complexes and FcγR on preosteoclasts. This pro-osteoclastogenic effect of low-sialylated IgG complexes was confirmed with natural immune complexes consisting of low- or high-sialylated anti-TNP IgG and TNP-26-BSA (Supplementary Fig. 3).

Analyses by dynamic light scattering revealed similar particle sizes of native and desialylated aggregated IgG (Supplementary Fig. 4) excluding size differences as a reason for the strong osteoclastogenic effect observed with the desialylated immune complexes. In addition, all IgG samples had been verified to be free of endotoxin to avoid endotoxin-mediated side effects.

### Immune complexes bind to FcγRII and III on preosteoclasts

**Figure 2:**
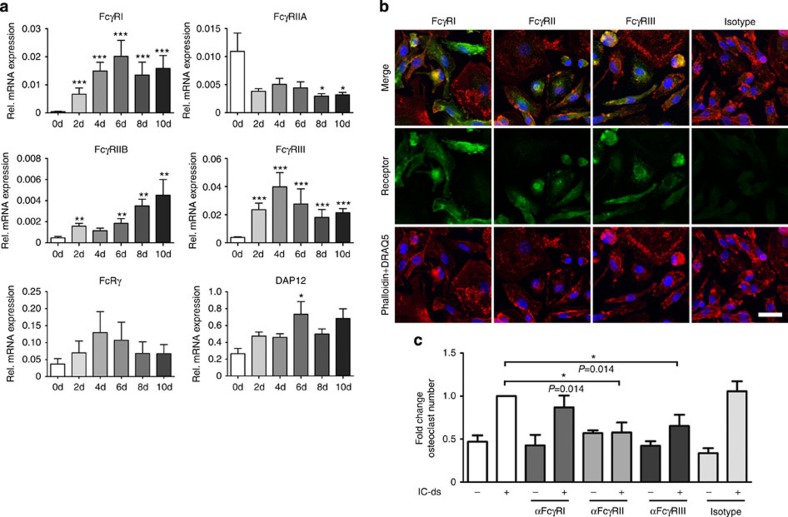
FcγRII and III mediate the signal of non-sialylated immune complexes on preosteoclasts. (**a**) Quantitative RT-PCR for relative mRNA levels of FcγR and adaptor proteins normalized on β2-microglobulin during osteoclastogenesis. Day 0 represents the stage of monocytes, day 6–8 the stage of late preosteoclasts and day 10 the stage of mature osteoclasts. Bars show mean±s.e.m. of three independent experiments. (**b**) Fluorescence microscopy images of FcγR expression on human preosteoclasts. FcγR are depicted in green, phalloidin staining for actin is depicted in red and DRAQ5 staining for the nuclei is depicted in blue. Scale bar, 25 μm (representative images of three independent experiments). (**c**) Fold change of osteoclast number after stimulation of preosteoclasts with 100 μg ml^−1^ of desialylated immune complexes (IC-ds) in the presence of 10 μg ml^−1^ of blocking antibodies against indicated FcγR. TRAP-positive cells with ≥3 nuclei were considered as osteoclasts. Bars show mean±s.e.m. of four independent experiments. Statistical analysis was performed with Mann–Whitney *U*-test. *P<0.5, **P<0.01, ***P<0.001.

Next, we investigated how the distinct FcγR are regulated during the process of human osteoclastogenesis. Therefore, we isolated mRNA from osteoclast cultures every second day from the stage of monocytes (baseline, day 0) to the stage of mature osteoclasts (day 10). We observed an early upregulation of FcγRI and III as well as of the two immunoreceptor tyrosine-based activation motif-domain-harbouring accessory proteins FcRy and DAP12 (Fig. 2a). In contrast, the inhibitory FcγRIIB seemed to be mainly expressed by mature osteoclasts. The expression of FcγRIIA halved during the first 2 days of culture but remained stable for the rest of the culture period. To further test which FcγRs are expressed on the protein level at the preosteoclast stage (day 6–8), we performed immunofluorescence stainings on preosteoclasts. Confirming the mRNA data, all FcγRs could be detected (Fig. 2b), though we could not discriminate between FcγRIIA and FcγRIIB as the antibody against FcγRII recognizes both. Interestingly, smaller preosteoclasts exhibited the brightest expression signal, whereas larger, more mature cells that started to form podosomes seemed to have a less pronounced FcγR expression.

To determine which FcγR is responsible for the observed pro-osteoclastogenic effect of desialylated immune complexes, we added blocking antibodies against FcγRI, FcγRII and FcγRIII during the stimulation of preosteoclasts with desialylated immune complexes. Blocking of either FcγRII or FcγRIII resulted in a virtually complete abrogation of the pro-osteoclastogenic activity of desialylated immune complexes (Fig. 2c), indicating that a concerted interaction of those FcγRs is needed to provide the signal for enhanced osteoclast development. In contrast, blocking of FcγRI had no effect, confirming previous reports about the dispensability of the high-affinity FcγR in osteoclast development and arthritis^19^,^23^.

### Desialylated immune complexes affect osteoclasts *in vivo*

We next wanted to investigate the implications of desialylated immune complexes on osteoclastogenesis *in vivo*. In patients with rheumatoid arthritis, immune complexes have been found in the synovial fluid of the joint space^24^,^25^,^26^. To simulate this situation we decided to inject murine-aggregated IgG into the knee joints of C57BL/6 mice. Again, a part of the IgG had been enzymatically desialylated before heat aggregation. The control group was injected with PBS. The injections were repeated after 4 days. After additional 3 days, bones were dissected and analysed for the number and size of osteoclasts in the trabecular bone adjacent to the injection site.

**Figure 3:**
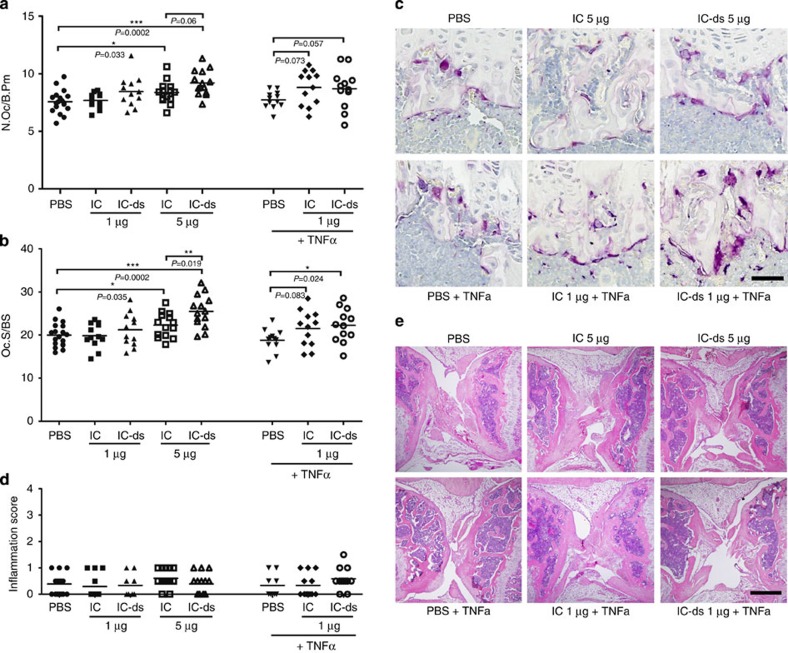
Non-sialylated immune complexes stimulate osteoclastogenesis *in vivo*. (**a**) Number of osteoclasts per bone perimeter (N.Oc/B.Pm) and (**b**) osteoclast surface per bone surface (Oc.S/BS) in tibial bone of C57BL/6 mice with intra-articular injection of the indicated amounts of native or desialylated (ds) murine immune complexes. Mice were injected at day 0 and day 4 with bone dissection at day 7. When indicated, 100 ng of TNFα was added to the first injection. Bars show mean±s.e.m. of ≥12 knee joints of three independent experiments. (**c**) Representative images of TRAP-stained tibial sections. Scale bar, 50 μm. (**d**) Inflammation score of haematoxylin-eosin stained joint sections with a score of 0 representing a healthy joint and a score of 4 representing a completely destroyed joint. Bars show mean±s.e.m. of ≥12 knee joints of three independent experiments. (**e**) Representative images of haematoxylin-eosin stained tibial sections. Scale bar, 500 μm. Statistical analysis was performed with Mann–Whitney *U*-test. *P<0.5, **P<0.01, ***P<0.001.

Mice that were injected with 5 μg of desialylated immune complexes (IC-ds) displayed a significantly higher osteoclast number and surface compared with PBS-injected mice (Fig. 3a–c). The injection of 5 μg of untreated (sialylated) immune complexes (IC) resulted in a mild increase in osteoclast number and surface, but the effect was much less pronounced compared with desialylated immune complexes, confirming our *in vitro* data. Notably, this difference vanished after co-injection of 100 ng of TNFα. In this case, both aggregates were able to induce osteoclastogenesis already at an amount of 1 μg.

Haematoxylin-eosin staining revealed no differences in the degree of inflammation between the groups with only localized, if any, inflammation around the small injury of the injection site (Fig. 3d,e). Hence, the observed pro-osteoclastogenic effect of desialylated immune complexes was independent of inflammatory tissue infiltration.

### IgG glycosylation and bone loss in arthritis patients

To address to which degree IgG Fc sialylation affects human bone architecture, we measured the amount of Fc sialylation, galactosylation and fucosylation of total IgG and disease-specific autoantibodies (ACPA) in 30 patients with rheumatoid arthritis and related these results to bone microstructures of the distal radius measured by micro-computed tomography (μCT). We grouped the patients into tertiles with a low, medium or high degree of Fc sialylation, galactosylation and fucosylation of IgG, and compared bone volume, trabecular numbers and trabecular thickness between these groups.

**Figure 4:**
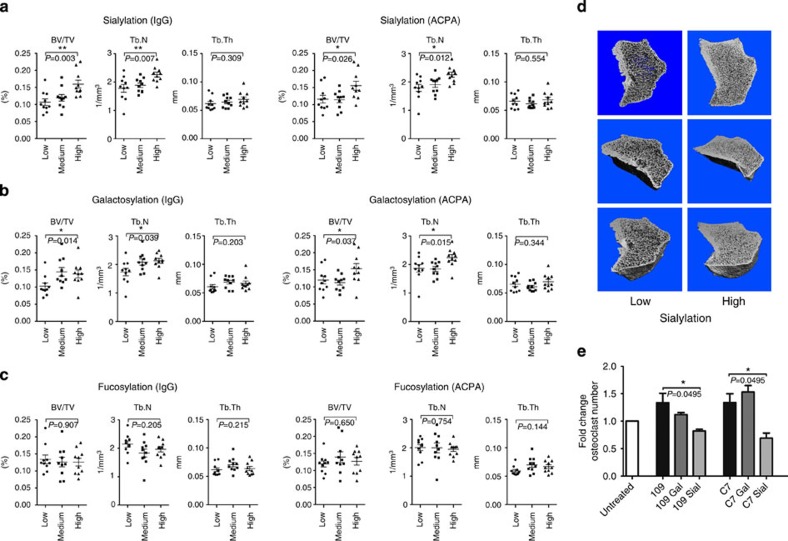
Sialylation status of IgG and ACPA control bone mass in rheumatoid arthritis patients. (**a**–**c**) Bone morphometric parameters including bone volume per tissue volume (BV/TV), trabecular number (Tb.N) and trabecular thickness (Tb.Th) of RA patients with low, medium or high levels of Fc (**a**) sialylation, (**b**) galactosylation or (**c**) fucosylation of total IgG and ACPA. Bars show mean±s.e.m. of10 patients in each tertile. Cutoffs for tertiles are as follows: IgG Fc sialylation (low: <14%, middle: 15–17.5%, high: >17.5%), ACPA Fc sialylation (<12%; 12–16%; >16%), IgG Fc galactosylation (<43.5%; 43.5–51%; >51%); ACPA Fc galactosylation (<44%; 44–54.5%; >54.5%); IgG Fc fucosylation (<85%; 85–87%; >87%); ACPA Fc fucosylation (<93%; 93–96%; >96%). (**d**) Two-dimensional (upper panel) and 3-dimensional (lower panels) reconstruction of the bone architecture of patients with low and high degree of Fc sialylation; (**e**) Fold change of osteoclast number after treatment of preosteoclasts for 72 h with 10 μg ml^−1^ of native, galactosylated (gal) or galactosylated and sialylated (sial) monoclonal ACPA (clone 109 and C7). TRAP-positive cells with ≥3 nuclei were considered as osteoclasts. Bars show mean±s.e.m. of three independent experiments. Statistical analysis was performed with Kruskal–Wallis test with Dunn’s correction (**a**–**c**) and Mann–Whitney *U*-test (e). *P<0.5, **P<0.01.

In accordance with our previous experiments, patients exhibiting IgG with low-sialylated or low-galactosylated Fc portions displayed a significantly decreased bone volume compared with patients with high levels of IgG Fc sialylation or galactosylation (Fig. 4a,b,d). This effect seems mainly be related to lower trabecular numbers, as no difference was observed in trabecular thickness. Similar results were obtained for disease-specific ACPA. The analysis of Fc fucosylation of IgG and ACPA did not show any relation to the bone structure (Fig. 4c). In addition, there was no difference between the groups regarding other parameters potentially impacting bone architecture or IgG glycosylation status^27^, such as age, sex, disease duration, disease activity or treatment status (Supplementary Table 1), suggesting that the observed differences in bone structure were confined to IgG sialylation and galactosylation.

To further define the role of IgG sialylation in human bone structure, we addressed whether Fc sialylation influences the bone phenotype even before the clinical onset of disease. We thereby made use of the observation that autoantibodies emerge several years before the onset of rheumatoid arthritis and that in some individuals bone changes precede the clinical onset of the disease^28^,^29^. When investigating IgG and ACPA Fc sialylation as well as bone structure in autoantibody-positive healthy individuals before the onset of rheumatoid arthritis, we again found that bone volume and trabecular numbers are significantly lower in individuals with low IgG and low ACPA sialylation compared with those with a high-sialylation status (Supplementary Fig. 5).

### Sialylated ACPA lose their pro-osteoclastogenic activity

To determine whether the observed association between IgG Fc galactosylation and bone volume is due to a direct effect of IgG Fc galactosylation or simply the fact that galactose is needed for the attachment of sialic acid to the IgG glycan, we enzymatically galactosylated and sialylated two human monoclonal ACPA (clones 109 and C7) (Supplementary Fig. 6) that have been generated from B cells from the joints of rheumatoid arthritis patients^30^. In consistence with previously reported data^20^, both clones promoted osteoclast differentiation (Fig. 4e). Sialylation of these antibodies resulted in a loss of their pro-osteoclastogenic activity to the point of even a slight inhibition of osteoclastogenesis. In contrast, galactosylation alone, without sialylation, did not seem to have an effect on the osteoclastogenic properties of the antibodies. In accordance with these findings, analysis of the sialic acid content per galactose residue on IgG in rheumatoid arthritis patients showed a significant association of higher sialylation rates with increased bone volume and trabecular numbers (Supplementary Fig. 7), suggesting that IgG galactosylation is of much less importance for osteoclastogenesis and bone loss than IgG sialylation.

### *N*-acetylmannosamine treatment protects mice from CIA

On the basis of these findings, we hypothesized that an increase of IgG sialylation protects from autoimmune-mediated bone loss. To test this hypothesis, we supplemented the drinking water of DBA/J1 mice exposed to CIA with the sialic acid precursor *N*-acetylmannosamine (ManNAc). ManNAc is rapidly metabolized into 5-*N*-acetylneuraminic acid, which is the most abundant mammalian sialic acid in the body^31^. Supplementation of ManNAc is effective in treating certain myopathies caused by enzymatic defects associated with protein hyposialyation, which is currently evaluated in clinical studies^32^.

**Figure 5:**
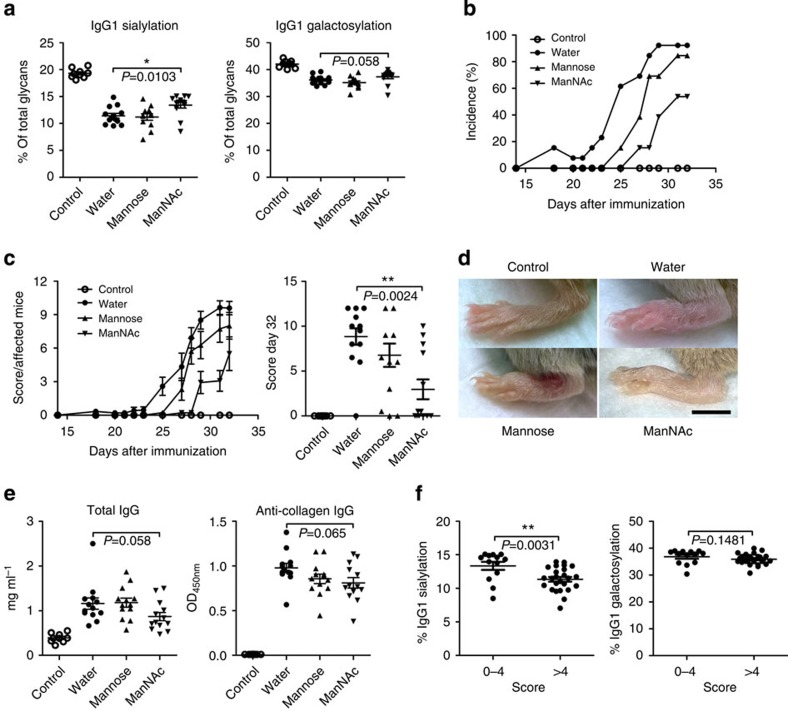
Treatment with ManNAc increases sialylation of IgG1 and reduces susceptibility to CIA. (**a**) Quantification of total serum IgG1 Fc sialylation and galactosylation at day 32 in non-induced control mice and mice induced for CIA receiving treatment with water, 10 g l^−1^ mannose or 10 g l^−1^ ManNAc. (**b**) Incidence of CIA. (**c**) Arthritis scores. (**d**) Representative images of hind paws at day 32. Scale bar, 5 mm. (**e**) Quantification of total serum IgG and collagen- specific IgG at day 32. Bars show mean±s.e.m. of combined data from two independent experiments (non-induced control group: *n*=9 mice; all other groups: *n*=13 mice). (**f**) Quantification of total serum IgG1 Fc sialylation and galactosylation at day 32 from mice that stayed healthy or developed mild arthritis (score 0–4) and from mice that developed severe arthritis (score >4). Bars show mean±s.e.m. of combined data from all mice subjected to CIA (score 0–4: *n*=13; score >4: *n*=25). Statistical analysis was performed with Mann–Whitney *U*-test. *P<0.5, **P<0.01.

When using a similar protocol, mice fed with 10 g l^−1^ ManNAc displayed a significantly higher IgG1 Fc sialylation compared with control mice that received either water or 10 g l^−1^ mannose (Fig. 5a). There was also a slightly higher IgG1 Fc galactosylation in ManNAc-treated mice, but this was not significant. ManNAc treatment led to lower incidence, delayed onset and lower arthritis scores compared with controls challenged with either water or mannose (Fig. 5b–d). No significant difference in the amount of total IgG and collagen-specific antibodies between the groups was observed, suggesting that ManNAc treatment did not generally impair the IgG-based immune response (Fig. 5e). Furthermore, IgG Fc sialylation, but not galactosylation, was significantly higher in mice that developed no or mild arthritis than in mice that developed severe arthritis (Fig. 5f), suggesting that higher IgG Fc sialylation levels reduce the susceptibility to experimental arthritis.

**Figure 6:**
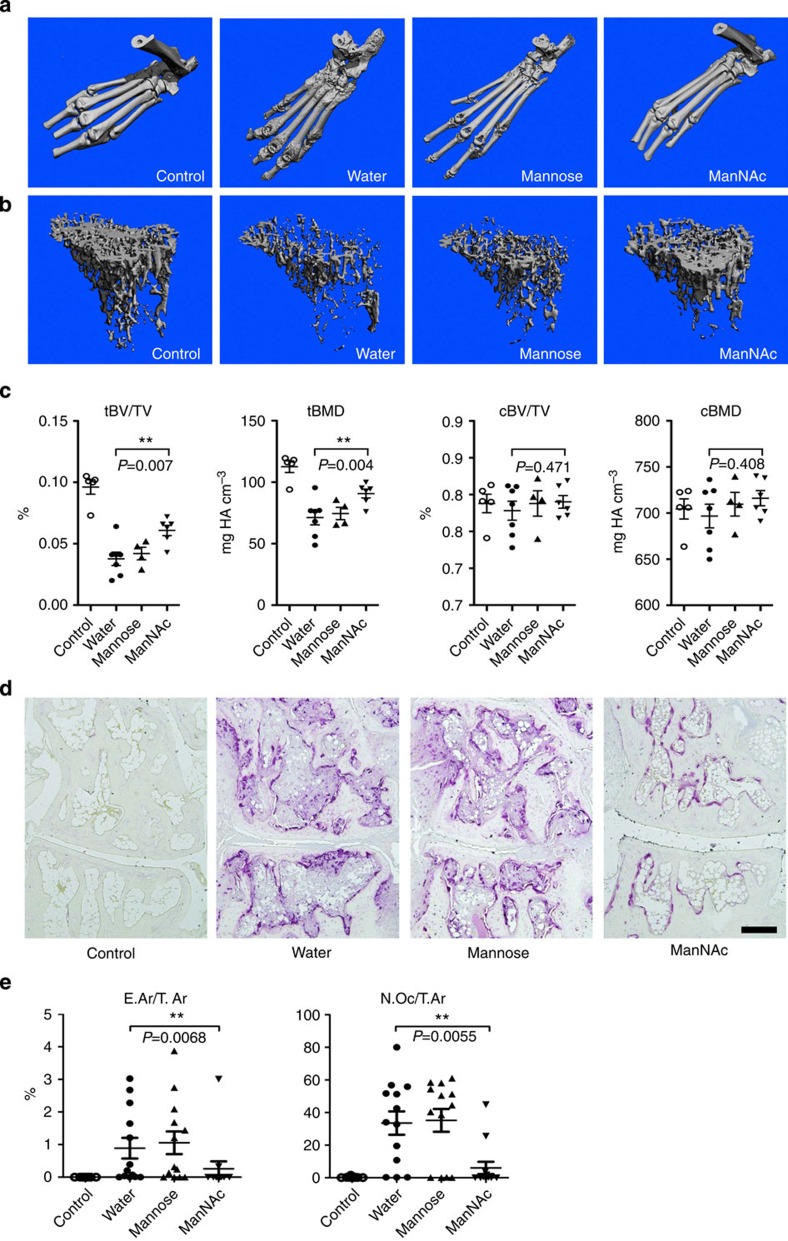
Treatment with *N*-acetylmannosamine (ManNAc) inhibits arthritis-mediated bone loss. (**a**,**b**) Representative 3-dimensional reconstructions of the bone architecture of hind paws (**a**) and tibial bones (**b**) of non-induced control mice and mice induced for collagen-induced arthritis (CIA) treated with water, 10 g l^−1^ mannose or 10 g l^−1^ ManNAc. (**c**) Bone morphometric parameters of the tibiae including trabecular and cortical bone volume per tissue volume (tBV/TV; cBV/TV) and trabecular and cortical bone mineral density (tBMD; cBMD). Bars show mean±s.e.m. from one representative experiment (non-induced control group: *n*=5 mice; water treated group: *n*=7 mice; all other groups *n*=6 mice). (**d**) Representative images of TRAP-stained paw sections. Scale bar, 200 μm. (**e**) Histological parameters of the hind paws including eroded area per tissue area (E.Ar/T.Ar) and osteoclast number per tissue area (N.Oc/T.Ar). Bars show mean±s.e.m. of combined data from two independent experiments (non-induced control group: *n*=9 mice; all other groups: *n*=13 mice). Statistical analysis was performed with Mann–Whitney *U*-test. **P<0.01.

Most importantly, ManNAc treatment was very effective to protect from local and systemic bone loss. μCT measurements on the hind paws and the tibial bones revealed that ManNAc-treated mice did virtually not develop bone erosions and experienced less severe systemic bone loss during CIA compared with controls exposed to either water or mannose (Fig. 6a,b). Quantitative analysis showed that particularly trabecular bone loss was significantly less severe in mice treated with ManNAc than in controls (Fig. 6c). Moreover, protection from bone erosions in ManNAc-treated mice was accompanied by significant reduction in osteoclast numbers in the paws confirming the concept that higher IgG Fc sialylation impairs osteoclast differentiation and mitigates inflammatory bone loss (Fig. 6d,e). The protective effect of high-sialylated IgG against bone loss was further confirmed in an IgG transfer model, in which untreated or *in vitro* sialylated IgG from mice, previously immunized against methylated bovine serum albumin (mBSA), was transferred to naive mice (Supplementary Fig. 8). Challenge with mBSA into the knee joint of the recipient mice led to increased osteoclast numbers only in mice that received untreated IgG. In contrast, mice receiving sialylated IgG were protected from enhancement of osteoclastogenesis, although they developed joint swelling to a similar extent as mice receiving untreated IgG.

## Discussion

In the last years, there have been several studies with controversial findings regarding the effects of immune complexes on osteoclastogenesis^17^,^19^,^33^. While the crosslinking of FcγR was found to be favourable for osteoclastogenesis^19^, it was unclear if the binding of immune complexes has the same effect. Here we show that the degree of IgG sialylation is of major importance for immune complex–osteoclast interactions and that only low- or non-sialylated immune complexes drive osteoclastogenesis *in vitro* as well as *in vivo*. Using random IgG, we further demonstrated that the pro-osteoclastogenic effect of non-sialylated immune complexes is not restricted to distinct antigen specificities but a common feature of all IgG antibodies.

In human serum only a minority of IgG of less than 20% is sialylated^5^. This low amount of sialylation suggests that the observed difference in preosteoclast stimulation between sialylated and non-sialylated immune complexes is not merely due to a reduced binding of sialylated IgG to the classical FcγR, but more likely due to an active suppression by sialylated IgG for example by binding to C-type lectins, such as dendritic cell immunoreceptor and dendritic cell-specific intercellular adhesion molecule-3-grabbing non-integrin, which recognize sialylated IgG and are expressed on myeloid cells^9^,^10^.

Interestingly, the putative suppressive function of sialylated IgG seems to be abrogated by strong pro-inflammatory stimuli, as, in our mouse model, after co-injection of 100 ng of TNF-α also the sialylated immune complexes promoted osteoclastogenesis.

When investigating the IgG Fc glycans of rheumatoid arthritis patients, we found a significant correlation between the Fc sialylation levels of random IgG as well as disease-specific autoantibodies (ACPA) and bone architecture. Patients with low levels of IgG Fc sialylation displayed lower bone volumes and lower trabecular numbers, while other parameters, such as age, sex or disease duration, did not differ among the groups. ACPA are well established as a major risk factor for the development of rheumatoid arthritis and are associated with a stronger disease course and enhanced bone erosion^34^,^35^. Of note, ACPA have been reported to be less sialylated than random IgG^21^, which may explain their strong pathogenicity. We now provided evidence that this feature may also be a reason for the direct pro-osteoclastogenic effect of ACPA that has been published earlier^20^. Indeed, ACPA that were sialylated *in vitro* completely lost their capacity to drive osteoclastogenesis.

We have also found a significant correlation between IgG Fc galactosylation and bone architecture in rheumatoid arthritis patients. But, as *in vitro* galactosylation of ACPA did not alter their pro-osteoclastogenic activity, IgG galactosylation does not seem to play a major role for direct immune complex–preosteoclast interactions. However, we cannot exclude indirect effects of IgG galactosylation on osteoclastogenesis for example by influencing the overall joint inflammation.

On the basis of our data we hypothesized that an enhancement of IgG sialylation may be a treatment strategy to inhibit autoimmune-mediated bone loss. To test this hypothesis, we used the sialic acid precursor ManNAc, which is an interesting approach as it can be taken up orally, has been shown to affect myopathies related to hyposialylation^32^ and enhances overall protein sialylation^36^. Indeed, mice induced for CIA that were fed with ManNAc displayed a significant increase in IgG sialylation compared with mice that received water or a mannose solution. Treatment with ManNAc not only resulted in a mitigated course of arthritis, but also blocked inflammatory osteoclastogenesis and bone erosion. Whether such an approach is effective in the treatment of rheumatoid arthritis and emerges as a more feasible treatment approach than costly intravenous immunoglobulin infusions, which have shown efficacy in the treatment of rheumatoid arthritis^37^,^38^, however, remains to be determined.

In summary, our data show that, apart from the regulation of immune effector functions, IgG sialylation controls osteoclast differentiation and bone mass in mice and humans pointing to a new link between the adaptive immune system and bone with direct relevancy for human disease.

## Methods

### Desialylation and deglycosylation of IgG and IC generation

Human IgG was taken from Beriglobin (Behring). Murine IgG was obtained from pooled serum of healthy C57BL/6 mice (Charles River) of different age and sex by purification over a protein G column (GE Healthcare) according to the manufacturer’s instructions. For desialylation, 1 mg of human or murine IgG was incubated with 5U or 10,000U neuraminidase (NEB) for 24 h or 48 h, respectively, at 37 °C. For deglycosylation, human IgG was incubated with 500 U mg^−1^ PNGase F (NEB) for 24 h at 37 °C. The efficiency of the enzymatic digestion was tested with a lectin blot. The digested IgG was purified over a protein G column (GE Healthcare) according to the manufacturer’s instructions and tested for endotoxin contamination using a LAL chromogenic endotoxin quantitation kit (Thermo scientific). Protein concentration was determined with the DC protein assay (Bio-Rad) and adjusted to 10 mg ml^−1^. Immune complexes were obtained by heat aggregation of the IgG at 63 °C for 30 min.

### Galactosylation and Sialylation of monoclonal antibodies

Monoclonal ACPA from the clones 109 and C7 and anti-TNP antibodies were generated as described elsewhere^30^,^39^. For galactosylation, 1 mg of IgG was incubated with 10 μM UDP-galactose (Calbiochem) and 2,5 mU of β1-4 galactosyltransferase (Sigma) in 50 mM MOPS, pH7.2 with 20 mM MnCl_2_ for 48 h at 37 °C. For sialylation, 1 mg of IgG was incubated with 10 μM CMP-sialic acid (Calbiochem) and 10 mU of α2-6 sialyltransferase (Sigma) in 50 mM MES, pH 6,0 with 10 mM MnCl_2_ for 48 h at 37 °C. The reactions were confirmed with a lectin blot.

### Lectin blotting

IgG was resolved on a sodium dodecyl sulfate–PAGE (SDS–PAGE) gel under reducing conditions, transferred to PVDF membranes and blocked with 3% deglycosylated gelatine (Sigma). Blots were incubated with biotinylated sumbuccus nigra lectin (2 μg ml^−1^) for sialic acid, erythrina cristagalli lectin (5 μg ml^−1^) for galactose or lens culinaris agglutinin (5 μg ml^−1^, all vector laboratories) for the detection of the core glycan, followed by incubation with an alkaline phosphatase conjugated mouse-anti-biotin antibody (Sigma) and detection with 4-nitro blue tetrazolium chloride/5-bromo-4-chloro-3-indolyl phosphate (Roche).

### Dynamic light scattering measurements

Angular-dependent dynamic light scattering was performed using a 6,328 nm-Laser and a CGS3-Goniometer (ALV) at a temperature of 20 °C with two avalanche diode detectors and an ALV-5000 correlator with 320 channels under cross-correlation. A range of scattering angels of 30°<θ<150° was covered in 10° steps. Intensity autocorrelation functions were transferred into electric field autocorrelation functions via Siegert relation and then transformed into the distribution of relaxation times *A*(*τ*) via regularized Laplace transformation. Resulting mean relaxation times for each angle were transferred into the apparent diffusion coefficient *D*_app_, which was then extrapolated to zero scattering angle (scattering vector square); the extrapolated result subsequently gave the hydrodynamic radius *R*_H_ through Stokes–Einstein relationship.

### Generation and stimulation of preosteoclasts

Human monocytes were purified by plastic adhesion of peripheral blood mononuclear cells that had been isolated from EDTA-blood of normal healthy donors using a Ficoll gradient (Lymphoflot, BioRad). Preosteoclasts were generated in α-Mem (Invitrogen) containing 10% fetal bovine serum (Biochrome) and 1% penicillin/streptomycin (Invitrogen) with 30 ng ml^−1^ M-CSF, 10 ng ml^−1^ RANKL and 1 ng ml^−1^ TGF-β (all Peprotech). After 6–9 days (depending on the donor), first binucleated cells appeared and preosteoclasts were incubated as indicated with the different IgG preparations. Osteoclast differentiation was evaluated by staining for TRAP using a Leukocyte Acid Phosphatase Kit (Sigma) according to the manufacturer’s instructions.

For the resorption assay, human preosteoclasts were generated and stimulated with IgG complexes on plates coated with calcium phosphate (Corning) under the same conditions as described above. Resorption was visualized with a von Kossa staining. In brief, we lysed the cells with water and incubated the wells for 30 min with 5% silver nitrate. After extensive washing, the silver stain was developed for 1 min with 5% sodium carbonate in 25% formaldehyde and unreacted silver was removed with 5% sodium thiosulfate for 5 min. For evaluation, photos were taken and the percentage of the resorbed area was calculated with Adobe Photoshop CS5 extended.

### Blocking of Fcγ receptors

Preosteoclasts were generated as described above and incubated for 1 h with 10 μg ml^−1^ of blocking antibodies against FcγRI (10.1, biolegend), FcγRII (AT10, abcam), FcγRIII (3G8, provided from the group of Falk Nimmerjahn) or isotype control (mouse IgG1, κ, biolegend), respectively. Subsequently, 100 μg ml^−1^ of desialylated aggregated IgG was added for 6 h, followed by a complete medium exchange. After additional 18 h, osteoclast differentiation was evaluated by staining cells for TRAP using a Leukocyte Acid Phosphatase Kit (Sigma) according to the manufacturer’s instructions.

### Immunofluorescence staining

Preosteoclasts were fixed with 4% PFA (pH 7.2) in PBS for 10 min at 37 °C. Fixed samples were then permeabilized and blocked for 1 h with 3% BSA in 0.1% Triton X-100 in PBS (all Sigma). Next, samples were incubated for 90 min with primary antibodies against FcγRI (12.7 μg ml^−1^, 10.1. Abcam), FcγRII (1:200, Abcam), FcγRIII (2 μg ml^−1^, J5511, Abcam) followed by 1 h incubation with the alexa-fluor-488 conjugated secondary antibodies (goat-anti-mouse for FcγRI and III and goat-anti-rabbit for FcγRII, 10 μg ml^−1^) together with alexa-fluor-568-labelled phalloidin (1:200). Samples were then incubated with DRAQ5 (all Life technologies) and mounted with FluorSave reagent (Calbiochem). Fluorescence images were acquired with a Zeiss confocal microscope.

### RNA isolation and quantitative RT-PCR analysis

Osteoclasts were differentiated from human monocytes as described above. Directly after monocyte isolation or after various times of culture, total RNA was extracted with peqGOLD TriFast (Peqlab) according to the manufacturer’s instructions. RNA was transcribed into cDNA using oligo(dT) primers and MuLV reverse transcriptase (Roche). Quantitative real-time PCR was performed with SYBR Green I-dTTP (Eurogentec) and the following primer pairs: β2-microglobulin (β2-MG) forward 5′- GATGAGTATGCCTGCCGTGTG -3′ and β2-MG reverse 5′- CAATCCAAATGCGGCATCT -3′, *fcγr1* forward 5′- GTGTCATGCGTGGAAGGATA -3′ and *fcγr1* reverse 5′- GCACTGGAGCTGGAAATAGC -3′, *fcγr2a* forward 5′- CCAGCATGGGCAGCTCTTCACC -3′ and *fcγr2a* reverse 5′- TGGGCAGCCTTCACAGGATCA -3′, *fcγr2b* forward 5′- GCGGCCATTGTTGCTGCTGTA -3′ and *fcγr2b* reverse 5′- AGAGCATCCGGGTGCATGAGA -3′, *fcγr3* forward 5′- ACAGGTGCCAGACAAACCTC -3′ *fcγr3* reverse 5′- TTCCAGCTGTGACACCTCAG -3′, *fcrγ* forward 5′- TGATTCCAGCAGTGGTCTTGCTCT -3′ and *fcrγ* reverse 5′- ATGCAGGCATATGTGATGCCAACC -3′, *dap12* forward 5′- CAGCGACCCGGAAACAGCGT -3′ and *dap12* reverse 5′- CGGCCTCTGTGTGTTGAGGTCG -3′.

### Intraarticular injection of immune complexes

All animal experiments were approved by the government of Mittelfranken, Germany. Male 8–week- old C57BL/6-mice (Charles River) were randomly allocated into groups and administered with an intraarticular injection through the patellar tendon of 5 μl of PBS containing the indicated amounts of murine immune complexes. The injection was repeated after 4 days. After additional 3 days, mice were killed and the bones were dissected for histological analysis.

### Induction of CIA and treatment with *N*-acetylmannosamine

Chicken collagen type II (4 mg ml^−1^; Sigma) was emulsified in equal amounts with complete Freunds adjuvant (Sigma) containing 5 mg ml^−1^ heat-inactivated *Mycobacterium tuberculosis* (H37Ra; Difco). For the induction of CIA, male 7-week-old DBA/1J mice (Janvier) were injected s.c. at the base of the tail with 100 μl of this emulsion. Mice were rechallenged after 21 days. *N*-acetylmannosamine was constantly administered to the drinking water at a concentration of 10 g l^−1^, beginning with the primary immunization. Control mice received water or water containing 10 g l^−1^ mannose (all Sigma). The allocation of the mice into the different groups was performed randomly. Clinical arthritis was evaluated every second day using a scoring system from 0 (no swelling) to 3 (severe swelling and erythema) for every limb, resulting in a maximally possible score of 12 per animal.

### Antigen-induced arthritis transfer model

mBSA (2 mg ml^−1^; Sigma) was emulsified in equal amounts with complete Freunds adjuvant (Sigma) containing 5 mg ml^−1^ heat-inactivated *Mycobacterium tuberculosis* (H37Ra; Difco). Male 6-week-old Balb/c mice (Janvier) were injected s.c. with 100 μl of this emulsion and simultaneously injected *i.p.* with 5 × 10^8^ heat-inactivated *Bordtella pertussis* (National Institute for Biological Standards and Control (NIBSC)) at day 0 and 7. After 21 days, the mice were challenged with 100 μg mBSA into one knee joint. At day 28, blood was taken and IgG was isolated with a protein G column (GE Healthcare) according to the manufacturer’s instructions. For sialylation, 1 mg of IgG was incubated with 750 μM CMP-sialic acid (Calbiochem) and 30 mU of α2-6 sialyltransferase (Sigma) in 100 mM Tris/HCl, pH 8,0 with 1 mM MnCl_2_ for 4 days at 37 °C. The reaction was confirmed with a lectin blot. For the IgG transfer, 2 mg of untreated or sialylated AIA-IgG was injected i.v. into naive male 9-week-old Balb/c (Janvier) mice. After 1 and 5 days, the right knee joints were injected with 100 μg mBSA. The left knee joints served as internal controls. Knee joint swelling was determined using a dial thickness gauge (Peacock) and expressed relative to the knee diameter at day 0. At day 8, mice were killed and the bones were dissected for histological analysis.

### Measurement of IgG concentrations in mouse serum

The serum concentrations of IgG were measured with an enzyme-linked immunosorbent assay (ELISA) quantitation kit (Bethyl Laboratories). For the assessment of collagen-specific IgG, plates were coated with 10 μg ml^−1^ chicken collagen type II (Sigma) instead of the capture antibody.

### Histology

Bones were fixed for 6 h in 4% formalin and decalcified in EDTA (Sigma-Aldrich). Serial paraffin sections (2 μm) were stained for TRAP using a Leukocyte Acid Phosphatase Kit (Sigma) according to the manufacturer’s instructions or with haematoxylin-eosin. All analyses were performed using a microscope (Nikon) equipped with a digital camera and an image analysis system for performing histomorphometry (Osteomeasure; OsteoMetrics).

### Micro-computed tomography of mice

μCT analyses were performed with a SCANCO Medical μCT 40 scanner using the following parameters: voltage, 40 kV; X-ray current, 250 μA; exposure time, 5,000 ms/projection for 720 projections; matrix, 1,024 × 1,024; voxel size in reconstructed image, 9 μm. Images were analysed with SCANCO evaluation software for parameters at the metaphyses of the proximal tibiae: ratio of bone volume to total volume, trabecular number and trabecular thickness.

### Patients

Thirty rheumatoid arthritis patients were analysed in detail for (i) bone structure by micro-computed tomography, (ii) IgG and ACPA glycosylation status and (iii) their demographic and disease-specific characteristics. The methodology of bone structure and antibody glycosylation analysis is described below. Age and sex were collected as the main demographic parameters showing a standard population of rheumatoid arthritis patients with a mean (±s.e.m.) age of 52.8±2.8 years and a dominance of female patients (20 out of 30). As disease-specific parameters we analysed disease duration, disease activity and anti-rheumatic treatment (conventional versus biological disease modifying anti-rheumatic drug therapy (DMARD)) in all the patients. Patients had established rheumatoid arthritis with a mean disease duration of 4.6±0.9 years. Their disease activity was 3.5±0.2 units according to the disease activity score (DAS) 28, resembling moderate disease activity. Furthermore, both, patients with conventional DMARD (methotrexate) therapy (16 out of 30) and biological DMARD (tumour necrosis factor inhibitor) therapy (14 out of 30) were analysed. In addition to the 30 patients with rheumatoid arthritis, a small group of 12 healthy individuals with autoantibody positivity, but no arthritis, was analysed for bone architecture and antibody glycosylation status as well as to exclude potential effects related to inflammation. All analyses of human material were performed in full agreement with institutional guidelines and with the approval of the Ethical committee of the University Hospital Erlangen (permit # 248_13B). Informed consent and permission to use the obtained data for research were obtained from all subjects enroled in the study.

### Micro-computed tomography in patients

HR-pQCT measurements were performed by an XtremeCT scanner (Scanco, Switzerland) at the ultra-distal radius of the right arm using the manufacturer’s standard *in vivo* protocol. The reference line was set manually. The first CT-slice was 9.5 mm proximal to the reference line. For scanning, the hand was immobilized in a carbon fibre cast. An antero-posterior scout view was then used to determine the region of interest. A total of 111 slices (voxel size 82 μm) were carried out. Details of the HR-pQCT measurements of the distal radius have been previously described by Boutroy *et al*.^40^ All measurements were performed with the same software (Version 6.0) by two well-trained physicians. Daily cross-calibrations with standardized control phantoms (Moehrendorf, Germany) were conducted to standardize measurements. Bone microstructure including trabecular bone volume fraction (BV/TV), trabecular number (Tb.N, 1/mm) and trabecular thickness (Tb.Th, mm) were analysed.

### IgG isolation

Human ACPA-IgG and ACPA-depleted IgG were purified from 30 μl of serum. ACPA were isolated by antigen affinity chromatography using cyclic citrullinated peptide (CCP2)-coated beads prepared by mixing biotinylated CCP2 dissolved in PBS at 1 mg ml^−1^ with Neutravidin Plus UltraLink resin (Pierce, Thermo Scientific) for 1 h at room temperature (RT). Following extensive washing, the CCP2 beads were dissolved in PBS and loaded into an Of1100 96-well filter plate (Orochem). Prediluted serum samples were applied and binding of ACPA to the beads was allowed by shaking the plate at 600 r.p.m. for 2 h at RT. Following incubation, the plate was centrifuged for 2 min at 1,500 × *g* and the flow through was collected in a 96-deepwell storage plate (Thermo). Beads were washed with 200 μl PBS and 25 mM ammonium bicarbonate followed by ACPA elution using 100 mM formic acid at pH 2.5 (pro analysis for mass spectrometry; Merck). The elution fraction was directly neutralized with 2 M TRIS. IgG (flow-through) and eluted ACPA-IgG were further purified using CaptureSelect IgG-Fc (Hu) Affinity Matrix (life technologies). To this end, 20 μl of CaptureSelect affinity resin was loaded into a 96-well filter plate (Of1100, 0.7 ml per well, PE frit, Orochem) and washed three times with 200 μl PBS using a vacuum manifold (Millipore), before transferring the CCP2 flow-through/elution samples to the plate. The plate was then incubated on a multiwell plate shaker (1.5 mm orbit, VWR) for 1 h at 450 r.p.m. to facilitate the binding of antibodies to the beads. The latter were then washed three times with 200 μl PBS using vacuum before the elution of (ACPA)-IgG by adding 100 μl of 100 mM formic acid at pH 2.4 (pro analysis for mass spectrometry; Merck). Again, the plate was shaken for 5 min on a shaking platform at 450 r.p.m. and elution of the antibodies was performed by centrifuging 1 min at 500 × *g*. IgG of DBA/1J mice were isolated from 2 μl of serum (pre-diluted in 200 μl PBS) using 20 μl of Protein G-Sepharose Fast Flow resin (GE Healthcare) according to the protocol described above for IgG capturing using CaptureSelect IgG-Fc (Hu) Affinity Matrix.

### Fc-glycosylation analysis by nano-LC-ESI-MS

IgG and ACPA-IgG eluates were dried in a vacuum centrifuge and subjected to tryptic digest by adding 200 ng trypsin (sequencing grade, Promega) in 40 μl ammonium bicarbonate buffer followed by overnight incubation at 37 °C. Digested ACPA and IgG antibodies were separated and analysed on an Ultimate 3000 UPLC system (Dionex Corporation, USA) coupled to a quadrupole-TOF mass spectrometer (MS) (micrOTOF-Q or maXis ultra-high resolution QTOF; Bruker Daltonics)^41^. The samples were injected and concentrated on a C18 solid phase extraction trap column (Dionex Acclaim PepMap100, 5 mm × 300 μm i.d.) conditioned with 0.1% TFA (mobile phase A) for 1 min at 25 μl min^−1^. Sample separation was achieved on an Ascentis Express C18 nano-liquid chromatography column (50 mm × 75 μm i.d., 2.7 μm HALO fused core particles, Supelco) conditioned at 900 nl min^−1^ with mobile phase A after which the following gradients of mobile phase A and 95% acetonitrile (mobile phase B) were applied: 0 min 3% B, 2 min 6% B, 4.5 min 18% B, 5 min 30% B, 7 min 30% B, 8 min 1% B and 11 min 1% B for human IgG; 0 min 3% B, 2 min 6% B, 4.5 min 18% B, 6 min 30% B, 8 min 30% B, 9 min 1% B and 12 min 1% B for mouse IgG. The UPLC was interfaced to the MS with a standard ESI source (Bruker Daltonics) and a sheath-flow ESI sprayer (capillary electrophoresis ESI-MS sprayer; Agilent Technologies)^41^. Mass spectra were recorded from *m/z* 600 to 2,000 with two averages at a frequency of 0.5 hz. To reduce glycan decay during ion transfer, the quadrupole ion energy and collision energy of the MS were set at 2 and 4 eV, respectively. The total analysis time per sample was 13 and 14 min for human and mouse IgG samples, respectively. IgG Fc-glycopeptides were identified based on their retention times and their accurate monoisotopic masses of double protonated and triple-protonated charged ions (human IgG1: EEQYN*STYR, mouse IgG1: EEQFN*STFR and mouse IgG2A: EDYN*STLR; N* stands for an N-glycosylated asparagine). Internal calibration of LC-MS spectra was performed in Bruker DataAnalysis 4.0 using a list of known glycopeptides, before exportation of the data to the mzXML format. A list of glycopeptide features, defined as a retention time window of ±15–20 s and a peak maximum within mass window of ±*m/z* 0.07, was extracted from each data set using the in-house developed ‘Xtractor2D or 3D’ software^41^. The extracted data, merged on a sample-data matrix, were finally evaluated using Microsoft Excel. Quality of mass spectra was determined based on intensities of total IgG1 glycoforms in both human and mice samples. The level of galactosylation, sialylation and fucosylation of human IgG1 were calculated using the following formulas: galactosylation=(G1F+G1FN+G1FS+G1FNS+G1+G1N+G1S)*0.5+G2F+G2FN+G2FS+G2FNS+G2+G2N+G2S;fucosylation=G0F+G1F+G2F+G0FN+G1FN+G2FN+G1FS+G2FS;sialylation=G1FS+G2FS+G1FNS+G2FNS+G1S+G2S (G: galactose, F: Fucose; N: bisecting N-Acetylglucosamine; S: sialic acid). The degree of galactosylation and sialylation of mouse IgG1and IgG2a was calculated using the following formulae: galactosylation=(G1+G1F+G1FN+G1FNeuAc+G1FNeuGc)*0.5+(G2F+G3F+G2FNeuGc+G3FNeuGc+G2FNeuGc2)*1);sialylation=(G1FNeuAc+G1FNeuGc+G2FNeuGc+G3FNeuGc)*0.5+G2FNeuGc2. (G: galactose, F: Fucose; N: bisecting N-Acetylglucosamine; NeuAc: N-Acetylneuraminic Acid; NeuGc: N-Glycolylneuraminic Acid).

### Data procession and statistical analysis

We performed computations and charts with GraphPad Prism 5.03 software. If not indicated differently, two-sided Mann–Whitney *U* test was used for statistical analysis (*P<0.05; **P<0.01; ***P<0.001). For comparison of glycosylation tertiles with respect to bone data in humans, the Kruskal–Wallis test with Dunn’s correction was used. We also performed a *post-hoc* power calculation for bone data in humans by taking into account the size of the observed effect and a type I error probability of 2.5% (adjusted for two tests, that is, low versus medium, low versus high), resulting in a power of 77.2% for the corresponding significant finding. Data are presented as mean±s.e.m. as well as medians and inter-quartile ranges. All analysis was performed in a blinded manner.

## Author contributions

U.H., M.H. and G.S. designed and performed experiments and analysed data. U.H., S.C.L., W.B. and K.D. performed animal experiments. R.P. and A.L. contributed to the enzymatic modification of IgG. S.F. and F.G. performed and analysed the dynamic light scattering measurements. K.A., V.M. and L.K. generated and provided monoclonal ACPA. Y.R., C.A.K., R.E.M.T. and H.U.S. isolated and analysed IgG from sera. H.B., G.K., F.N. and M.H. provided valuable material and intellectual input. U.H. and G.S. wrote the manuscript.

## Additional information

**How to cite this article:** Harre, U. *et al*. Glycosylation of immunoglobulin G determines osteoclast differentiation and bone loss. *Nat. Commun.* 6:6651 doi: 10.1038/ncomms7651 (2015).

## Supplementary Material

Supplementary Information Supplementary Figures 1-8 and Supplementary Table 1
