# Supplementary material for: Glycosylation of immunoglobulin G determines osteoclast differentiation and bone loss
Source: Nat Commun. 2015 Mar 31;6:6651. doi: 10.1038/ncomms7651 (PMC4389255; doi:10.1038/ncomms7651)
Supplement: Supplementary Information — Supplementary Figures 1-8 and Supplementary Table 1 [file ncomms7651-s1.pdf]

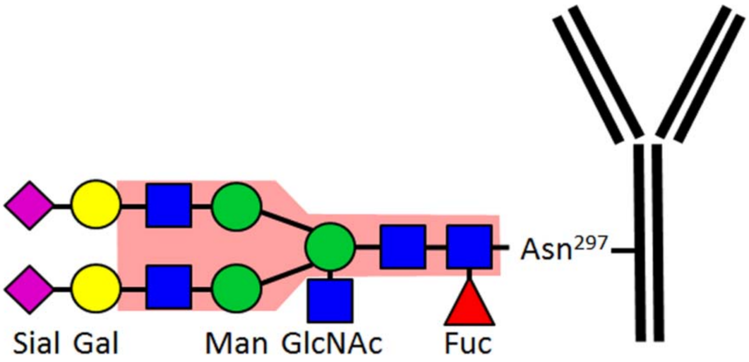

Supplementary figure 1 Schematic overview of the Fc-glycan of IgG. The glycan is composed of a constant core domain (highlighted in red) composed of mannose (Man) and N-acetylglucosamine (GlcNAc) residues to which galactose (Gal), sialic acid (Sial), fucose (Fuc), and GlcNAc can be additionally attached.

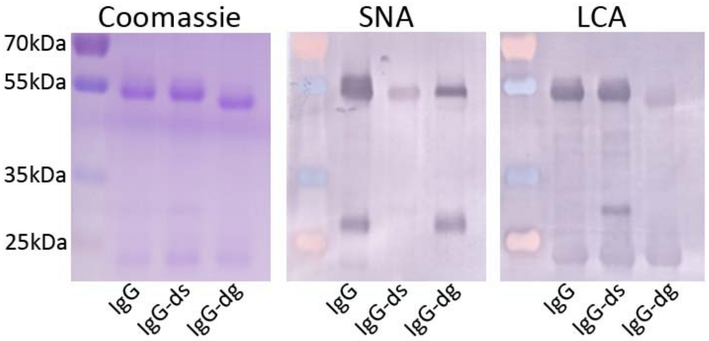

Supplementary figure 2 Verification of sugar removal from IgG. Desialylation (ds) and deglycosylation (dg) of IgG has been tested by lectin blotting with sambuccus nigra lectin (SNA) detecting sialic acid and lens culinaris lectin (LCA) detecting the core glycan. Coomassie staining was used as loading control.

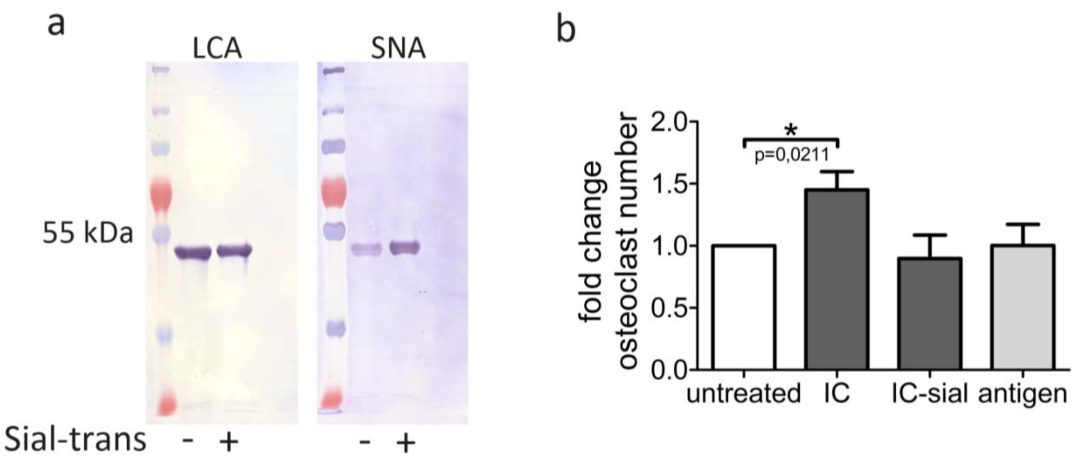

Supplementary figure 3 Low-sialylated antigen containing immune complexes promote osteoclastogenesis. (a) Lectin blot of anti-TNP IgG after the attachment of sialic acid with sialyltransferase (Sial-trans). Sialic acid was detected with sambuccus nigra lectin (SNA). Detection of the core glycan with lens culinaris agglutintin (LCA) was used as loading control. (b) Fold change of osteoclast number after treatment of preosteoclasts for 72h with 10µg/ml of immune complexes consisting of low-sialylated (IC), or high-sialylated (IC-sial) anti-TNP IgG and TNP-26-BSA. Treatment with the antigen alone served as negative control. TRAP positive cells with ≥3 nuclei were considered as osteoclasts. Bars show mean ± s.e.m. of 4 independent experiments. Statistical analysis was performed with with Mann-Whitney U test. *p<0.05.

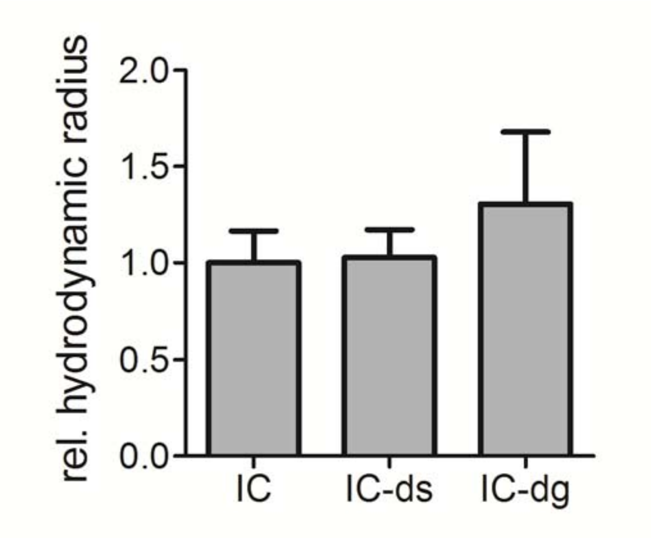

Supplementary figure 4 Changes in particle size of immune complexes. Dynamic light scattering measurements revealed no differences in particle size of immune complexes generated by heat aggregation of native, desialylated (ds) or

deglycosylated (dg) IgG. Shown are relative hydrodynamic radii that were normalized on the mean radius of untreated IgG complexes (IC). Bars show mean ± s.e.m. of 3-5 independent experiments (IC, IC-ds: n=5; IC-dg: n=3).

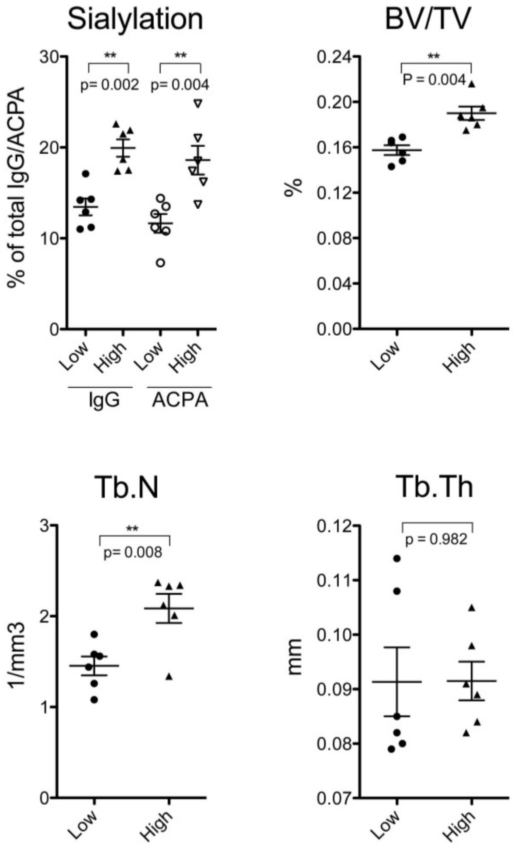

Supplementary figure 5 IgG and ACPA sialylation determine bone structure in preclinical disease. Bone morphometric parameters were measured in autoantibody-positive healthy individuals at risk for developing rheumatoid arthritis (n=12 individuals). (a) Fc sialylation of total IgG and ACPA. Cutoffs for low vs. high Fc sialylation were 17,2% and 14,1% for IgG and ACPA, respectively. (b) Bone volume per tissue volume (BV/TV), (c) trabecular number (Tb.N) and (d) trabecular thickness (Tb.Th) in individuals with low and high Fc sialylation status. Bars show mean ± s.e.m. of 6 individuals per group. Statistical analysis was performed with with Mann-Whitney U test. **p<0.01.

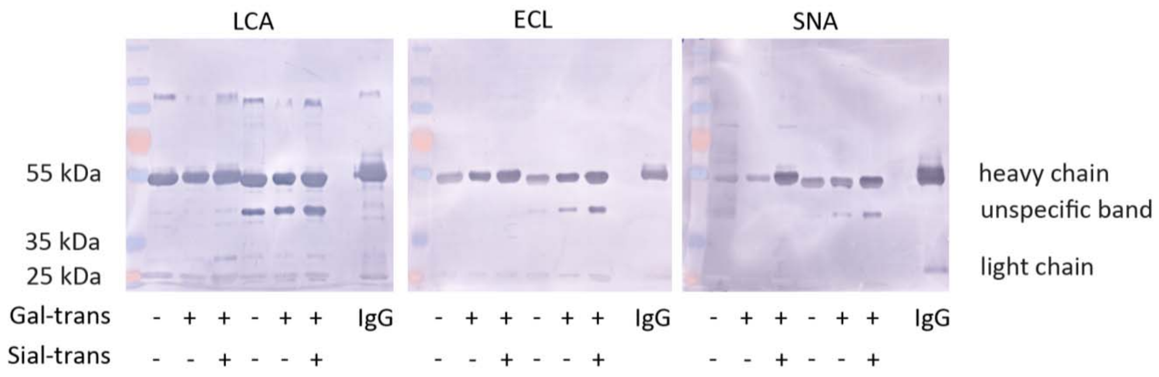

Supplementary figure 6 Verification of the addition of sugar moieties to monoclonal ACPA. The enzymatic attachment of galactosis (Gal-trans) and sialic acid (Sial-trans) to monoclonal ACPA (clones 109 and C7) has been tested by lectin blotting with erythrina cristagalli lectin (ECL) detecting galactose, and sambuccus nigra lectin (SNA) detecting sialic acid. Detection of the core glycan with lens culinaris agglutintin (LCA) was used as loading control. Pooled human serum IgG served as a reference for the glycosylation.

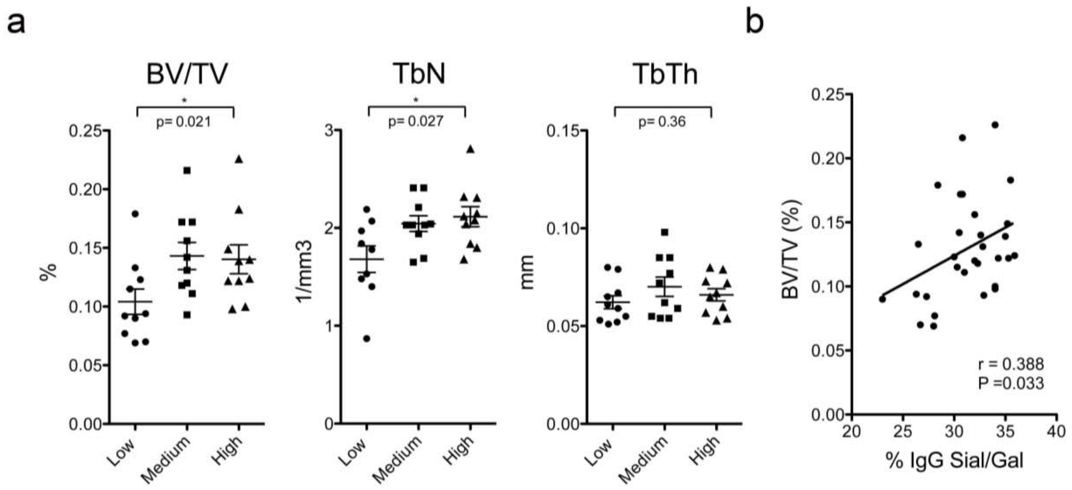

Supplementary figure 7 Relation between sialylation and galactosylation and bone mass. (a) Bone morphometric parameters including bone volume per tissue volume (BV/TV), trabecular number (Tb.N) and trabecular thickness (Tb.Th) of RA patients with low, medium or high ratio of Fc sialylation to galactosylation of IgG (n=10 patients per tertile). Cutoffs for tertiles for Fc sialylation/galactosylation ratio are: low: <30.5%, middle: 31-32.5%, high: >32.5%). Bars show mean ± s.e.m. of 10 individuals per tertile. (b) Correlation between bone volume per tissue volume (%, y-axis) and Fc sialylation/galactosylation ratio of IgG (%, x-axis). Statistical analysis was performed with Kruskal Wallis test with Dunn's correction (a) and Spearman's rho (b). *p<0.05.

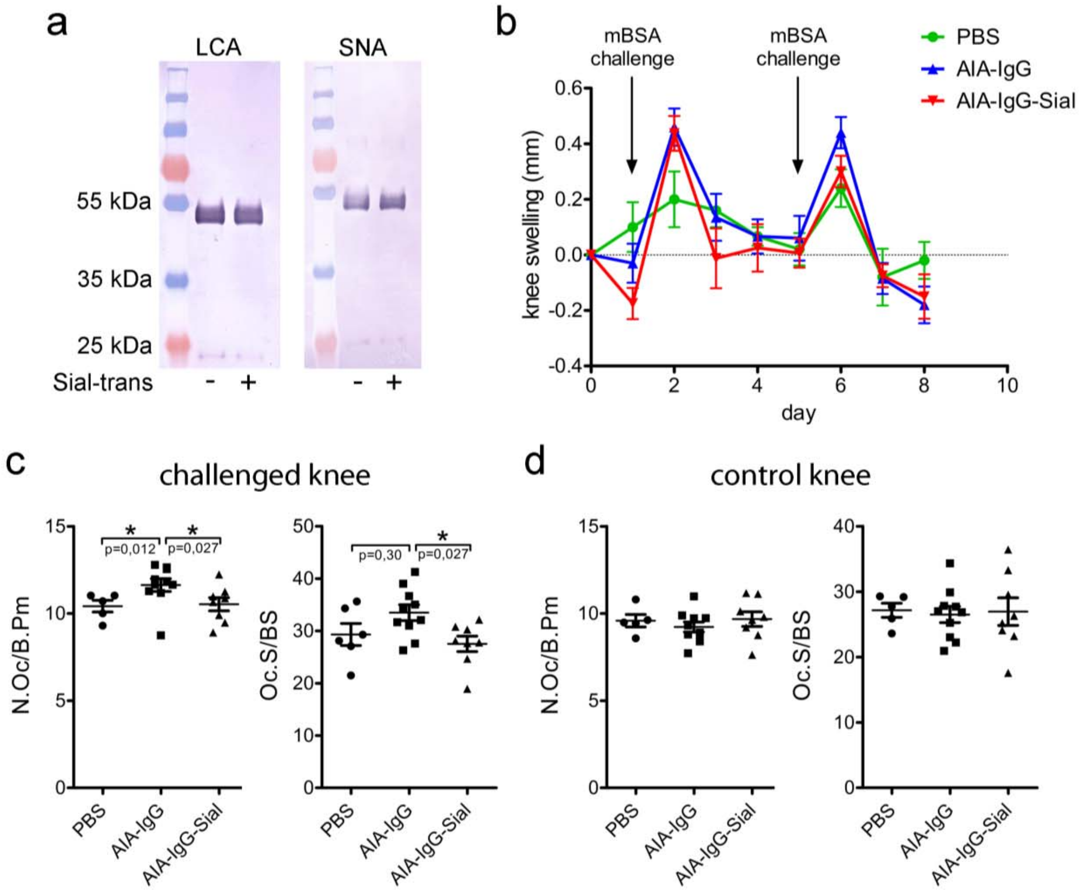

Supplementary figure 8 Elevated IgG sialylation protects mice from inflammatory bone loss in an antigen induced arthritis transfer model. (a) Lectin blot of untreated and *in vitro* sialylated (Sial-trans +) serum IgG (AIA-IgG) of Balb/c mice immunized against methylated bovine serum albumin (mBSA). IgG sialylation was detected with sambuccus nigra lectin (SNA). Detection of the core glycan with lens culinaris agglutintin (LCA) was used as loading control. (b) Swelling of the challenged knee joints of Balb/c mice during the experiment. At day 0, mice were injected *i.v.* with 2mg of native or sialylated AIA-IgG. At day 1 and day 5, the knee joint was challenged with 100µg of mBSA. (c)+(d) Number of osteoclasts per bone perimeter (N.Oc/B.Pm) and osteoclast surface per bone surface (Oc.S/BS) in tibial bone of the challenged (c) and the control (d) knee joints. Bars show mean ± s.e.m. of combined data from two independent experiments (PBS: n=5 mice; AIA-IgG: n=10 mice; AIA-IgG-Sial: n=8 mice). Statistical analysis was performed with with Mann-Whitney U test. *$p<0.05$.

	ACPA Low	ACPA Medium	ACPA High	p	IgG Low	IgG Medium	IgG High	p
**Age (years)**								
Sialylation	50.8±4.2	55.6±5.2	52.2±5.7	0.85	49.2±5.4	55.2±4.6	54.2±5.1	0.68
Galactosylation	51.4±5.7	53.0±4.5	54.2±5.1	0.87	52.1±5.6	55.2±4.6	51.3±5.1	0.92
Fucosylation	51.7±4.7	54.9±5.0	55.0±8.2	0.93	50.2±5.4	55.2±4.6	53.2±5.2	0.88
**Sex (N females)**								
Sialylation	7/10	7/10	6/10	0.86	6/10	7/10	7/10	0.68
Galactosylation	7/10	7/10	6/10	0.86	6/10	7/10	7/10	0.86
Fucosylation	7/10	7/10	6/10	0.86	6/10	7/10	7/10	0.86
**Duration (years)**								
Sialylation	5.8±1.8	3.8±1.3	4.3±1.5	0.52	5.5±1.9	3.9±1.3	4.5±1.5	0.65
Galactosylation	5.1±1.9	3.9±1.3	4.9±1.4	0.83	6.1±1.9	3.4±1.2	4.5±1.5	0.42
Fucosylation	5.5.±1.9	4.0±1.3	4.5±1.5	0.76	5.4±1.9	3.9±1.3	4.6±1.5	0.80
**DAS28 (units)**								
Sialylation	3.5±0.3	3.4±0.4	3.6±0.3	0.91	3.6±0.3	3.4±0.4	3.6±0.3	0.93
Galactosylation	3.3±0.3	3.3±1.3	3.9±0.3	0.45	3.4±0.4	3.4±1.2	3.5±0.3	0.90
Fucosylation	3.4.±0.4	3.7±0.4	3.6±0.3	0.72	3.4±0.3	3.3±0.3	3.8±0.4	0.54
**Biological Use (N)**								
Sialylation	4/10	5/10	5/10	0.87	4/10	5/10	5/10	0.87
Galactosylation	4/10	6/10	4/10	0.59	4/10	5/10	5/10	0.87
Fucosylation	3/10	5/10	6/10	0.40	5/10	5/10	4/10	0.87

Supplementary table 1. Clinical characteristsics of the rheumatoid arthritis patients according to tertiles for different antibody glycosylation. Data for the 3 different tertiles (low, medium, high) for antibody glycosylation are shown. Values indicate means ± s.e.m.. P-values were calculated by ANOVA. ACPA, anti-citrullinated protein antibodies, IgG, immunoglobulin G. DAS28, disease activity score 28.
